# Pre-Conception Physical Activity and the Risk of Gestational Diabetes Mellitus: Findings from the BORN 2020 Study

**DOI:** 10.3390/nu17111832

**Published:** 2025-05-28

**Authors:** Antigoni Tranidou, Antonios Siargkas, Ioannis Tsakiridis, Emmanuela Magriplis, Aikaterini Apostolopoulou, Georgia Koutsouki, Michail Chourdakis, Themistoklis Dagklis

**Affiliations:** 1 3rd Department of Obstetrics and Gynecology, School of Medicine, Faculty of Health Sciences, Aristotle University of Thessaloniki, 541 24 Thessaloniki, Greece; antigoni.tranidou@gmail.com (A.T.); antonis.siargkas@gmail.com (A.S.); igtsakir@auth.gr (I.T.); koutsoukig@gmail.com (G.K.); 2 Department of Food Science and Human Nutrition, Agricultural University of Athens, Iera Oos 75, 118 55 Athens, Greece; emagriplis@eatsmart.gr; 3 Laboratory of Hygiene, Social & Preventive Medicine and Medical Statistics, School of Medicine, Faculty of Health Sciences, Aristotle University of Thessaloniki, 541 24 Thessaloniki, Greece; katapost@yahoo.gr (A.A.); mhourd@auth.gr (M.C.)

**Keywords:** physical activity, IPAQ, pre-pregnancy, nutrition, Mediterranean diet, gestational diabetes mellitus, GDM

## Abstract

**Background/Objectives**: Pre-conception health behaviors may influence the risk of gestational diabetes mellitus (GDM), but evidence on the joint effects of physical activity (PA) and dietary patterns remains limited. This study investigated the associations between pre-conception PA and GDM risk and explored their interaction with adherence to a Mediterranean diet (MD). **Methods**: This analysis used data from the BORN2020 cohort, which included pregnant women in Greece (2020–2022). Pre-conception PA was assessed using the International Physical Activity Questionnaire-Short Form (IPAQ-SF), expressed as the metabolic equivalent of task (MET)-min/week and categorized into quartiles. Adherence to the MD was assessed via the Trichopoulou score and then grouped into tertiles. Multivariable logistic regression models were computed, accounting for sociodemographic and clinical covariates, including sedentary time and post-lunch nap frequency. **Results**: In total, 524 women were included and 13.9% (*n* = 73) were diagnosed with GDM. Women who developed GDM were significantly older (mean age 34.41 vs. 31.98 years, *p* < 0.0001), were more likely to be >35 years old (46.6% vs. 26.6%, *p* < 0.001), had higher pre-pregnancy BMI (median 24.6 vs. 22.7 kg/m^2^, *p* = 0.014), and were more likely to be obese (23.3% vs. 11.8%, *p* = 0.012). No significant association was observed between total pre-conception PA and GDM risk. Compared to the lowest PA quartile, women in the medium (aOR = 0.80, 95% CI: 0.45–1.40), high (aOR = 1.12, 95% CI: 0.52–2.39), and very high (aOR = 1.10, 95% CI: 0.50–2.38) PA quartiles showed no significant differences in GDM risk. PA, when modeled as a continuous variable, showed no significant trend (aOR = 0.99, 95% CI: 0.99–1.00; *p*-trend = 0.61). A joint analysis of PA and MD adherence also yielded no significant associations overall. However, in very small BMI-stratified subgroups, a low level of PA combined with very high MD adherence in normal-weight women was associated with increased GDM risk (aOR = 14.06, 95% CI: 1.55–165.54, *p* = 0.022), while in obese women, very high levels of PA and medium MD adherence showed a potentially protective effect (aOR = 0.006, 95% CI: 8.43 × 10^−6^–0.42, *p* = 0.048). These subgroup findings require cautious interpretation, due to the limited size of the sample set and wide confidence intervals. **Conclusions**: In this cohort, pre-conception PA, either alone or in combination with MD adherence, was not a reliable predictor of GDM. While our subgroup signals are hypothesis-generating, they do not yet support changes to clinical risk stratification. Future large-scale and interventional studies should investigate combined lifestyle interventions before conception to clarify the potential synergistic effects on GDM prevention.

## 1. Introduction

Gestational diabetes mellitus (GDM) is one of the most common complications of pregnancy and is associated with both short- and long-term adverse outcomes for both the mother and offspring, including pre-eclampsia, macrosomia, and an increased risk of future type 2 diabetes mellitus [1,2]. The increasing incidence of GDM has positioned it as a significant public health concern. The etiology of GDM is multifactorial, encompassing both genetic predispositions and modifiable environmental influences, such as lifestyle, dietary habits, and levels of physical activity [3]. Notably, insufficient physical activity and elevated pre-pregnancy body weight have been consistently identified as significant risk factors for the development of GDM [4,5]. Physical exercise is known to contribute to improved glucose metabolism, enhanced insulin sensitivity, and the maintenance of a healthy body weight, factors that are pivotal in both the prevention and management of GDM [6].

Modern lifestyles are increasingly sedentary, with adults spending over 8 h per day seated due to remote working, commuting, and screen-based leisure activities, and this prolonged sitting has been independently linked with impaired insulin sensitivity and higher GDM risk [7], an effect that is largely mediated by body mass index (BMI) but that persists even after adjustment in some analyses [8]. Among modifiable antenatal risk factors, physical activity (PA) has been proposed as a protective measure against GDM through its effects on glucose metabolism, insulin sensitivity, and weight regulation [6,9]. While several studies have consistently shown that higher levels of PA during pregnancy may reduce GDM risk [10,11], the evidence regarding the influence of PA before conception remains inconclusive [12]. Some prospective studies have reported inverse associations between pre-pregnancy PA and GDM risk [4,9,13], particularly among women at greater metabolic risk [14], while others have found no significant effect [3,7]. Among the available self-report instruments, the IPAQ-SF [15] has strong validation evidence in Greek adult populations and its brevity minimizes respondent burden; however, as a recall-based tool, it may misclassify intensity and duration and is susceptible to social desirability bias, potentially attenuating true associations. Notably, the emerging longitudinal data suggest that specific types of pre-conception activity, such as muscle-strengthening exercises, may confer a protective effect against GDM, even though total pre-pregnancy activity levels alone were not consistently associated with a reduced risk [5].

In addition, sleep patterns, both nocturnal and in the daytime, have been implicated in glucose regulation [16]. Shorter night-time sleep during early pregnancy is independently linked to higher GDM risk [17]. Moreover, earlier work studying non-pregnant adults suggests that habitual, long daytime naps (>1 h) may promote insulin resistance through altered cortisol secretion [18]. Similarly, adherence to a healthy dietary pattern, particularly the Mediterranean diet (MD), has been linked to a lower risk of GDM [19]. The MD is characterized by a high intake of fruits, vegetables, whole grains, legumes, and nuts, moderate fish and dairy intake, and a low level of consumption of red meat and processed foods. A number of studies suggest that pre-pregnancy adherence to the MD may independently reduce GDM risk [20,21].

Given that lifestyle behaviors often cluster, investigating joint effects is critical to better understanding cumulative risk or protective patterns. Using data from the BORN2020 prospective cohort, we investigated whether higher levels of pre-conception PA are associated with reduced GDM risk after adjustment for seated hours and post-lunch sleep and whether MD adherence modifies these associations, with additional stratification according to pre-pregnancy BMI as an effect modifier.

## 2. Materials and Methods

### 2.1. Study Design and Participants

The current analysis was conducted within the BORN2020 prospective cohort study (ethics decision no: 6.231/29 July 2020), which enrolled pregnant women attending the 3rd Department of Obstetrics and Gynecology, School of Medicine, Faculty of Health Sciences, Aristotle University of Thessaloniki, Greece between July 2020 and October 2022 [22]. Participants were recruited during their first routine perinatal visit, between 11^+0^ to 13^+6^ weeks of gestation. Inclusion criteria encompassed women aged 18 years or older, with singleton pregnancies, and without pre-existing diabetes mellitus. Exclusion criteria included multiple pregnancies, serious medical conditions (e.g., hypertension or renal disease), adherence to special diets (e.g., a vegetarian diet), and incomplete data.

### 2.2. Data Collection and Measured Variables

Baseline data, including maternal age, weight, sedentary time, nap frequency, physical activity data, height, medical and obstetric history, smoking habits, and conception via assisted reproductive technologies (ART), were collected through structured interviews by trained personnel. Pre-pregnancy weight and height data were used to calculate BMI according to standardized classification criteria [23]. Due to the small number of participants classified as underweight (*n* = 1), this subgroup was not included in analyses stratified according to BMI to avoid unreliable statistical estimates.

### 2.3. Physical Activity Exposure Assessment

PA was assessed using the International Physical Activity Questionnaire-Short Form (IPAQ-SF) [15], administered by trained doctors via structured interviews during the first perinatal visit at 11^+0^ to 13^+6^ weeks of gestation, with participants reporting their usual activity over the six months preceding conception. Total physical activity was calculated in metabolic equivalent of task (MET)-minutes/week as MET × minutes/day × days/week, with assigned MET values of 3.3 for walking, 4.0 for moderate-intensity activity, and 8.0 for vigorous-intensity activity, according to the IPAQ scoring guidelines. Total PA was computed as the sum of walking, moderate, and vigorous MET-minutes/week. Participants were categorized into quartiles, based on the distribution of total PA. A four-level categorical variable was also constructed to examine the joint associations between PA and dietary quality, combining low, medium, high, and very high PA quartiles with low and high MD adherence (based on the Trichopoulou score median split [24]). Additionally, PA was modeled as a continuous variable to assess linear trends in the risk of GDM (*p*-trend), using a multivariate logistic regression model.

### 2.4. Dietary Assessment

Dietary intake was evaluated using a locally validated semi-quantitative food frequency questionnaire (FFQ) [25], with nutrient intakes calculated through NutriSurvey software V. 2007. Adherence to the MD was assessed using the Trichopoulou score, and participants were categorized into quartiles [24].

### 2.5. Diagnosis of GDM

All participants between 24 and 28 weeks of gestation underwent a 75-g oral glucose tolerance test (OGTT), and GDM was defined according to the hyperglycemia and adverse outcomes (HAPO) criteria [26]: fasting plasma glucose ≥ 92 mg/dL (5.1 mmol/L), 1-h plasma glucose ≥ 180 mg/dL (10.0 mmol/L), or 2-h plasma glucose ≥ 153 mg/dL (8.5 mmol/L). A diagnosis of GDM was made if any one of these thresholds was met or exceeded.

### 2.6. Statistical Methods

Regarding the population characteristics, for variables following a normal distribution, mean and SD are provided; otherwise, the median and quartiles are reported. The Shapiro–Wilk (<50 samples) or Kolmogorov–Smirnov (≥50 samples) tests were used to assess normality. The *p*-values were obtained using a *t*-test for normally distributed variables, a Mann–Whitney test for non-normally distributed variables, and a chi-squared test for categorical variables. Fisher’s exact test (*n* < 5) or a chi-squared test (*n* ≥ 5) was applied in the case of binary variables, contingent on the sample size.

A logistic regression model was fitted to estimate the association between PA and GDM risk. The model included adjustment for maternal age, pre-pregnancy BMI status, average seated hours per day, the frequency of post-lunch naps per day, smoking status, mode of conception (assisted reproductive technology), thyroid disease, total energy intake, and MD score (continuous).

Categorization of PA and MD adherence into quartiles, respectively, was based on established practice in the epidemiological literature for ease of interpretation and to reflect potential non-linear relationships with GDM risk. Similar cutoffs have been used in studies examining lifestyle factors and chronic disease risk (e.g., [27,28]). In our study, both PA and MD scores were also examined as continuous variables to complement the categorical analysis and assess linear trends across the full range. All analyses were conducted using complete case analysis. Observations with missing values in either the exposure, outcome, or covariates included in the model were excluded.

The joint effects of PA and MD adherence were assessed by including the four-level combined variable in the regression model, with the “Low PA + Low MD” group serving as the reference. Formal interaction terms (e.g., PA × BMI group) were not included in our models. Instead, we constructed joint exposure variables that categorized participants into mutually exclusive subgroups, based on combined levels of physical activity and Mediterranean Diet adherence (e.g., “High PA + Low MD”). These variables allowed us to assess the combined association of lifestyle factors with GDM risk but did not constitute formal interaction terms within a single regression model.

Stratified analyses were also conducted according to pre-pregnancy BMI, using adjusted logistic models within each subgroup. All analyses were performed using R software (version 4.2.1). A *p*-value of <0.05 was considered statistically significant.

## 3. Results

### Participant Characteristics

Of 797 women who met the eligibility criteria, 524 completed the full version of the IPAQ and were included in the final analysis; among these, 73 women (13.9%) developed GDM. Baseline characteristics according to GDM status are presented in Table 1. Those women who developed GDM were significantly older (mean age 34.41 ± 4.80 years vs. 31.98 ± 4.88 years, *p* < 0.0001) and were more likely to be aged over 35 years (46.58% vs. 26.61%, *p* < 0.001), compared to those without GDM. Pre-pregnancy BMI was also significantly higher in women with GDM (median 24.6 kg/m^2^ vs. 22.7 kg/m^2^, *p* = 0.014), and obesity prevalence (BMI ≥ 30 kg/m^2^) was more than double among GDM cases (23.29% vs. 11.75%, *p* = 0.012). Rates of overweight were similar between groups. Although smoking before pregnancy appeared more frequently in the GDM group (12.33% vs. 8.43%), the difference was not statistically significant (*p* = 0.39). There were no significant differences in total pre-conception physical activity, adherence to the Mediterranean diet, ART conception, or thyroid disease between GDM and non-GDM groups.

In the adjusted model, no significant association was observed between pre-conception physical activity and the risk of GDM (Table 2). Specifically, compared to women in the lowest quartile of PA (reference group), those in the medium PA quartile had an aOR of 0.80 (95% CI: 0.45–1.40, *p* = 0.46), those in the high PA quartile had an aOR of 1.12 (95% CI: 0.52–2.39, *p* = 0.76), and those in the very high PA quartile had an aOR of 1.10 (95% CI: 0.50–2.38, *p* = 0.81); none of these associations reached statistical significance. In addition, when total PA was analyzed as a continuous variable (MET-min/week), no significant trend was observed (aOR = 0.99, 95% CI: 0.99–1.00, *p*-trend = 0.61).

The joint associations of pre-conception physical activity and Mediterranean diet adherence with GDM risk are presented in Table 3. Women with both low PA levels and low MD adherence served as the reference group.

Among those with low PA levels, none of the combinations with higher MD adherence were significantly associated with GDM risk. Specifically, the adjusted odds ratios (aORs) for GDM were 0.74 (95% CI: 0.20–2.87, *p* = 0.65) for low MD + medium MD, 0.13 (95% CI: 0.00–7.26, *p* = 0.34) for low PA + high MD, and 2.55 (95% CI: 0.52–12.63, *p* = 0.24) for low PA + very high MD; all were non-significant.

In the medium PA group, aORs also remained non-significant across MD adherence levels: 1.57 (95% CI: 0.37–6.70, *p* = 0.53) for low MD adherence, 1.58 (95% CI: 0.46–5.95, *p* = 0.47) for medium MD adherence, 1.59 (95% CI: 0.33–7.32, *p* = 0.55) for high MD adherence, and 1.04 (95% CI: 0.12–6.11, *p* = 0.97) for very high MD adherence.

Among women with high PA, none of the MD combinations were statistically significant either. Compared to the reference group, the aORs were 1.61 (95% CI: 0.39–6.70, *p* = 0.50) for low MD adherence, 0.40 (95% CI: 0.07–1.86, *p* = 0.25) for medium MD adherence, 1.44 (95% CI: 0.29–6.67, *p* = 0.64) for high MD adherence, and 1.79 (95% CI: 0.35–8.59, *p* = 0.46) for very high MD adherence.

Lastly, in the very high PA group, none of the joint categories reached statistical significance. The aORs were 2.24 (95% CI: 0.61–8.93, *p* = 0.23) for low MD adherence, 0.86 (95% CI: 0.23–3.42, *p* = 0.86) for medium MD adherence, 0.30 (95% CI: 0.01–2.18, *p* = 0.30) for high MD adherence, and 0.64 (95% CI: 0.03–5.01, *p* = 0.71) for very high MD adherence.

Stratified analyses according to pre-pregnancy BMI are presented in Table 4. Among normal-weight women, compared to the low PA reference group, the aORs for GDM were 2.09 (95% CI: 0.82–5.44, *p* = 0.12) for medium PA, 0.71 (95% CI: 0.22–2.09, *p* = 0.54) for high PA, and 1.22 (95% CI: 0.43–3.37, *p* = 0.69) for very high PA, none of which were statistically significant. In overweight women, the aORs were 0.65 (95% CI: 0.09–4.29, *p* = 0.65) for medium PA, 1.44 (95% CI: 0.26–8.84, *p* = 0.67) for high PA, and 1.60 (95% CI: 0.28–10.30, *p* = 0.60) for very high PA, with all being non-significant. Among obese women, the medium PA group had an aOR of 2.18 (95% CI: 0.31–16.08, *p* = 0.43), the high PA group had an aOR of 1.63 (95% CI: 0.23–11.12, *p* = 0.61), and the very high PA group was associated with a non-significant aOR of 0.15 (95% CI: 0.00–1.57, *p* = 0.15). When total PA was treated as a continuous variable (MET-min/week), no significant trends were observed across the BMI subgroups: *p*-trend = 0.64 for normal-weight women, 0.59 for overweight women, and 0.18 for obese women.

Table 5 presents the joint associations between pre-conception physical activity, Mediterranean diet adherence, and GDM risk, stratified by pre-pregnancy BMI. Among normal-weight women, most PA–MD combinations were not significantly associated with GDM risk compared to the reference group (Low PA + Low MD). For example, those with Low PA + Medium MD had an aOR of 1.74 (95% CI: 0.29–14.24, *p* = 0.56), those with Low PA + High MD had an aOR of 0.94 (95% CI: 0.03–12.11, *p* = 0.94), and those with Low PA + Very High MD showed a statistically significant increased risk (aOR = 14.06, 95% CI: 1.55–165.54, *p* = 0.022). However, this estimate should be interpreted cautiously, given the wide confidence interval and the limited number of cases. This finding may reflect reverse causality or selection bias due to the small numbers involved, rather than reflecting a true protective or harmful effect. Medium PA combinations yielded non-significant associations across all MD adherence levels (e.g., Medium PA + High MD: aOR = 4.20, 95% CI: 0.55–39.75, *p* = 0.17). Similarly, among those in the High and Very High PA quartiles, none of the MD adherence levels showed significant associations. For instance, Very High PA + Very High MD had an aOR of 2.94 × 10^−7^ (95% CI: 2.69 × 10^−302^–5.69 × 10^24^, *p* = 0.99).

Among overweight women, all PA–MD combinations showed wide confidence intervals and *p*-values of ≥0.55. Notably, Low PA + Medium MD had an aOR of 0.14 (95% CI: 8.38 × 10^−5^–38.71, *p* = 0.55), while most other estimates were unstable or extreme (e.g., High PA + High MD: aOR = 1.48 × 10^7^, *p* = 1), which was likely due to sparse data in the subgroups. No statistically significant associations were detected.

In obese women, a borderline association was observed for Very High PA + Medium MD, which was associated with a reduced GDM risk (aOR = 0.006, 95% CI: 8.43 × 10^−6^–0.42, *p* = 0.048). Although this result reached conventional statistical significance, it should be interpreted cautiously, due to potential model instability and a small subgroup size. Other combinations, including High PA + Medium MD (aOR = 0.03, 95% CI: 2.06 × 10^−5^–2.08, *p* = 0.20) and Low PA + Very High MD (aOR = 0.02, 95% CI: 6.87 × 10^−5^–1.58, *p* = 0.13), also suggested protective trends, but did not reach statistical significance.

Notably, many aORs in the BMI-stratified joint analyses are extreme or unstable; several CIs span orders of magnitude, reflecting the very sparse cell counts in these subgroups. These estimates should, therefore, be regarded as exploratory. Power in these stratified analyses was limited, especially within the obese subgroup, where cell sizes were smallest; as such, null results in these groups do not exclude modest true effects.

## 4. Discussion

The main findings of this study were as follows: (1) no significant association was found between total pre-conception PA, whether categorized into quartiles or treated continuously, and GDM risk; (2) these null estimates persisted after controlling for age, BMI, sedentary behavior, nap frequency, and other established confounders; and (3) cross-classification of PA with MD adherence, as well as BMI-stratified models, revealed no significant joint effects. Collectively, these consistent null findings suggest that habitual exercise performed before conception, whether considered alone, in conjunction with dietary patterns, or within the BMI strata, does not substantially influence glycemic trajectories once pregnancy begins. Notably, several maternal characteristics were significantly associated with GDM risk: women who developed GDM were older and had a higher median pre-pregnancy BMI (24.6 vs. 22.7 kg/m^2^), with a notably higher prevalence of obesity (23.3% vs. 11.8%) compared to those without GDM.

Two exploratory interaction signals emerged when PA was cross-classified with adherence to the MD. Among normal-weight women, those reporting low levels of PA combined with very high MD adherence displayed a markedly elevated risk of GDM. This finding may suggest that dietary quality alone, in the absence of sufficient physical activity, may not confer a protective effect. Conversely, obese women engaging in very high levels of PA but with only medium MD adherence appeared substantially protected. Both estimates, however, were based on small subgroups and were characterized by wide confidence intervals, suggesting that these may be chance findings rather than clinically meaningful effects. Beyond these outliers, no coherent pattern of joint PA and dietary influence was observed across our many analyses.

Our findings are in accordance with earlier investigations that likewise failed to demonstrate an independent protective effect of pre-pregnancy activity. For example, Kiljan et al. reported an inverse association between leisure-time PA and an abnormal glucose-challenge test that disappeared after BMI adjustment (aOR 0.68; 95% CI 0.43–1.05) [3]. Similarly, Mendelian-randomization analyses similarly show no causal effect of genetically predicted moderate physical activity (OR 0.40; 95% CI 0.08–2.06), moderate to vigorous activity (OR 0.96; 95% CI 0.58–1.57), or accelerometer-based activity (OR 0.99; 95% CI 0.90–1.09) on GDM risk [7].

It is plausible that women who are highly active before conception reduce their activity once pregnant, which may be attributed to nausea, fatigue, or concerns about fetal safety, while maintaining caloric intakes that are calibrated to higher energy expenditure, thereby neutralizing any prior metabolic advantage [29]. In addition, the dose, intensity, and mode of exercise required to influence insulin sensitivity may need to exceed that captured by self-report instruments such as the IPAQ-SF. The measurement error inherent in questionnaire-based PA assessment would likely bias associations toward the null, while residual confounding by sleep quality, gestational weight gain, or in-pregnancy PA cannot be excluded.

This study has notable strengths. We focused on the pre-conception period, an arguably critical window for intervention that has not yet been as extensively studied as exercise during pregnancy. We also had access to detailed information on important covariates, including sedentary hours and nap frequency, which strengthens the validity of our findings by isolating the effect of physical activity. Importantly, we also included dietary quality. In particular, we adjusted for adherence to the MD diet pattern in the pre-pregnancy period, which is a known protective factor for GDM and related metabolic outcomes [30].

However, certain limitations should be acknowledged. Physical activity was assessed retrospectively at the first prenatal visit, introducing a potential recall bias. The IPAQ-SF, although validated, may misclassify activity intensity (e.g., vigorous vs. moderate activity) or capture muscle-strengthening exercises. Its recall-based format further risks misclassification, particularly regarding intermittent or structured exercise sessions, which could attenuate true associations if high-intensity or resistance activities are under-reported. MD adherence was not evaluated independently as a primary exposure but was only evaluated jointly with PA, whereas our prior work examined it independently and found that MD adherence was protective against GDM risk [21]. We also did not analyze data on physical activity during the pregnancy itself. It remains possible that women who maintain or increase their activity during gestation may reduce their GDM risk, but this was not assessed in this analysis, and it remains an important area for future research. Finally, despite the relatively large sample size, limited statistical power in stratified analyses, especially among obese women, may have masked small true effects. The generalizability of our findings may be limited to similar populations; the results from BORN 2020 may differ in other ethnic or socioeconomic groups with different baseline activity patterns or GDM prevalence. Finally, residual confounding cannot be entirely excluded. However, we did include major known confounders in our models to mitigate this concern. Overall, while our study contributes new data on an understudied exposure time frame, these limitations temper our interpretation and suggest that further research is needed to confirm our findings.

From a clinical standpoint, our findings do not support the use of pre-conception physical activity levels alone as a reliable predictor of GDM risk. While prior research has shown mixed results, sometimes finding benefits in specific subgroups, the overall evidence remains inconclusive. In the meantime, promoting regular physical activity throughout pregnancy remains a well-established and evidence-based recommendation for maternal health [31]. Women may modify their lifestyle behaviors once pregnancy is confirmed, reducing PA due to nausea or safety concerns while maintaining a caloric intake calibrated to pre-conception activity. Such shifts could mask any protective effects of pre-pregnancy exercise. Moreover, those noticing early dysglycemia may improve their diet or level of activity, introducing a reverse causality that biases subgroup estimates (e.g., extreme OR in normal-weight, low-PA/high-MD women).

Recent evidence reinforces the importance of integrated lifestyle approaches. A systematic review and meta-analysis of 116 trials (40,940 women) found that combined diet and PA programs reduced GDM incidence compared with the usual level of care (risk ratios 0.59–0.95 across delivery formats) [32]. Health programs integrating nutrition counseling and structured exercise support showed significant GDM risk reductions in overweight and obese pregnant women, compared to standard care [33].

The lack of a clear protective effect of pre-conception PA alone suggests that standalone exercise promotion before pregnancy may be insufficient for GDM prevention. Instead, multifaceted interventions, integrating both diet and structured PA, may be more effective. Health systems should prioritize early pregnancy or periconception programs that address multiple lifestyle behaviors concurrently, rather than focusing solely on pre-pregnancy exercise levels.

## 5. Conclusions

In this Greek prospective cohort study, pre-conception physical activity, either alone or in combination with MD adherence, was not significantly associated with the risk of developing gestational diabetes mellitus. Although subgroup signals emerged, they were based on very small numbers, showed wide confidence intervals, and should be regarded as hypothesis-generating only. Given these findings, future research should prioritize large, intervention-based studies that begin in the pre-conception period and follow women through pregnancy, incorporating objective PA assessment (e.g., accelerometry) alongside dietary and behavioral monitoring. Such rigorous designs will be essential to determine whether integrated lifestyle interventions can effectively reduce GDM risk across the continuum from before conception into gestation. 

## Figures and Tables

**Table 1:** Maternal characteristics of the study population, according to gestational diabetes mellitus status.

Variable	Non-GDM (*n* = 451)	GDM (*n* = 73)	*p*-Value
Maternal age (years), mean (SD)	31.98 (±4.88)	34.41 (±4.8)	<0.0001
Maternal age > 35 years (%)	120 (26.61%)	34 (46.58%)	<0.001
Pre-pregnancy BMI (kg/m^2^), median (Q1–Q3)	22.7 (20.9–26.4)	24.6 (21.7–29.4)	0.014
Obesity (BMI ≥ 30) (%)	53 (11.75%)	17 (23.29%)	0.012
Overweight (%)	149 (33.04%)	33 (45.21%)	0.058
Normal weight (%)	281 (62.31%)	38 (52.05%)	0.12
Smoking before pregnancy (%)	38 (8.43%)	9 (12.33%)	0.39
ART conception (%)	33 (7.32%)	7 (9.59%)	0.66
Thyroid disorder (%)	62 (13.75%)	7 (9.59%)	0.43
Total PA (MET-min/week), median (Q1–Q3)	1194 (594–2047)	1098 (594–1710)	0.60
MD score, median (Q1–Q3)	5 (3–6)	4 (3–6)	0.68

Student’s *t*-test for normally distributed continuous variables; Mann–Whitney U-test for non-normally distributed continuous variables; chi-squared or Fisher’s exact test for categorical variables. ART: assisted reproductive technology; BMI: body mass index; GDM: gestational diabetes mellitus; PA: physical activity; MET: metabolic equivalent of task.

**Table 2:** Association between pre-conception physical activity and the risk of gestational diabetes mellitus.

Exposure	aOR (95% CI)	*p*-Value
PA Low (reference)	1.00	-
PA Medium	0.8 (0.45, 1.4)	0.46
PA High	1.12 (0.52, 2.39)	0.76
PA Very high	1.1 (0.5, 2.38)	0.81
Total PA (continuous, MET-min/week)	0.99 (0.99, 1)	0.61 (*p*-trend)

Multivariable logistic regression, adjusted for maternal age, pre-pregnancy BMI, sedentary hours per day and frequency of post-lunch naps, smoking status, ART conception, thyroid disorder, total energy intake, and Mediterranean diet score. The *p*-trend was calculated using total physical activity as a continuous variable in the multivariable model. GDM: gestational diabetes mellitus; PA: physical activity; MET: metabolic equivalent of task.

**Table 3:** Joint associations of pre-conception physical activity (PA) and Mediterranean Diet (MD) adherence with gestational diabetes mellitus (GDM).

Group	Non-GDM	GDM	aOR (95% CI)	*p*-Value
Low PA + Low MD (reference)	36 (7.98%)	5 (6.85%)	1.00	-
Low PA + Medium MD	59 (13.08%)	7 (9.59%)	0.74 (0.2, 2.87)	0.65
Low PA + High MD	20 (4.43%)	2 (2.74%)	0.13 (0, 7.26)	0.34
Low PA + Very high MD	8 (1.77%)	5 (6.85%)	2.55 (0.52, 12.63)	0.24
Medium PA + Low MD	27 (5.99%)	5 (6.85%)	1.57 (0.37, 6.7)	0.53
Medium PA + Medium MD	40 (8.87%)	9 (12.33%)	1.58 (0.46, 5.95)	0.47
Medium PA + High MD	21 (4.66%)	4 (5.48%)	1.59 (0.33, 7.32)	0.55
Medium PA + Very high MD	13 (2.88%)	2 (2.74%)	1.04 (0.12, 6.11)	0.97
High PA + Low MD	23 (5.1%)	6 (8.22%)	1.61 (0.39, 6.7)	0.5
High PA + Medium MD	54 (11.97%)	3 (4.11%)	0.4 (0.07, 1.86)	0.25
High PA + High MD	19 (4.21%)	4 (5.48%)	1.44 (0.29, 6.67)	0.64
High PA + Very high MD	18 (3.99%)	4 (5.48%)	1.79 (0.35, 8.59)	0.46
Very high PA + Low MD	27 (5.99%)	8 (10.96%)	2.24 (0.61, 8.93)	0.23
Very high PA + Medium MD	50 (11.09%)	7 (9.59%)	0.86 (0.23, 3.42)	0.86
Very high PA + High MD	22 (4.88%)	1 (1.37%)	0.3 (0.01, 2.18)	0.3
Very high PA + Very high MD	14 (3.1%)	1 (1.37%)	0.64 (0.03, 5.01)	0.71

Multivariable logistic regression, adjusted for maternal age, pre-pregnancy BMI, sedentary hours per day, and frequency of post-lunch naps, smoking status, ART conception, thyroid disorder, total energy intake. GDM: gestational diabetes mellitus; PA: physical activity; MET: metabolic equivalent of task.

**Table 4:** Associations between pre-conception physical activity (PA) and gestational diabetes mellitus (GDM) risk, stratified by pre-pregnancy body mass index.

Exposure	Normal Weight	Overweight	Obese
	aOR (95% CI), *p*-Value	aOR (95% CI), *p*-Value	aOR (95% CI), *p*-Value
PA Low (reference)	1.00	1.00	1.00
PA Medium	2.09 (0.82, 5.44), *p* = 0.12	0.65 (0.09, 4.29), *p* = 0.65	2.18 (0.31, 16.08), *p* = 0.43
PA High	0.71 (0.22, 2.09), *p* = 0.54	1.44 (0.26, 8.84), *p* = 0.67	1.63 (0.23, 11.12), *p* = 0.61
PA Very high	1.22 (0.43, 3.37), *p* = 0.69	1.6 (0.28, 10.3), *p* = 0.6	0.15 (0, 1.57), *p* = 0.15
Total PA (continuous)	0.99 (0.99–1.00), *p*-trend = 0.64	1.00 (0.99–1.00), *p*-trend = 0.59	1.00 (0.99–1.00), *p*-trend = 0.18

Stratified multivariable logistic regression models were adjusted for maternal age, sedentary hours per day and frequency of post-lunch naps, smoking status, ART conception, thyroid disorder, total energy intake. GDM: gestational diabetes mellitus; PA: physical activity; BMI: body mass index.

**Table 5:** Joint associations of pre-conception physical activity and Mediterranean diet adherence with gestational diabetes mellitus risk, stratified by pre-pregnancy body mass index.

Exposure	Normal Weight	Overweight	Obese
	aOR (95% CI), *p*-Value	aOR (95% CI), *p*-Value	aOR (95% CI), *p*-Value
Low PA + Low MD (reference)	1.00	1.00	1.00
Low PA + Medium MD	1.74 (0.29, 14.24), *p* = 0.56	0.14 (8.38 × 10^−5^, 38.71), *p* = 0.55	0.05 (0, 2.33), *p* = 0.16
Low PA + High MD	0.94 (0.03, 12.11), *p* = 0.94	8.46 × 10^7^ (4.09 × 10^−114^, -), *p* = 1	8.69 × 10^−10^ (-, 1.08 × 10^134^), *p* = 1
Low PA + Very high MD	14.06 (1.55, 165.54), *p* = 0.022	4.91 × 10^7^ (4.73 × 10^−108^, -), *p* = 1	0.02 (6.87 × 10^−5^, 1.58), *p* = 0.13
Medium PA + Low MD	3.92 (0.4, 39.73), *p* = 0.22	5.47 × 10^9^ (2.82 × 10^−103^, -), *p* = 1	0.21 (0, 8.02), *p* = 0.44
Medium PA + Medium MD	4.07 (0.77, 32.28), *p* = 0.12	5.19 × 10^7^ (8.45 × 10^−103^, -), *p* = 1	0.26 (0, 22.22), *p* = 0.56
Medium PA + High MD	4.2 (0.55, 39.75), *p* = 0.17	1.21 × 10^7^ (5.88 × 10^−115^, -), *p* = 1	0.32 (0, 13.44), *p* = 0.58
Medium PA + Very high MD	2.44 (0.09, 34.69), *p* = 0.52	0.58 (9.78 × 10^−76^, 1.12 × 10^73^), *p* = 1	0.2 (0, 9.03), 0.45
High PA + Low MD	1.76 (0.18, 17.27), *p* = 0.61	7.59 × 10^7^ (7.3 × 10^−108^, -), *p* = 1	5.93 × 10^−10^ (-, 5.92 × 10^111^), *p* = 1
High PA + Medium MD	1.35 (0.40–4.62), *p* = 0.62	2.23 (6.02 × 10^−78^, 5.34 × 10^76^), *p* = 1	0.03 (2.06 × 10^−5^, 2.08), *p* = 0.2
High PA + High MD	1.4 × 10^−7^ (5.44 × 10^−247^, 1.51 × 10^19^), *p* = 0.99	1.48 × 10^7^ (6.13 × 10^−97^, -), *p* = 1	1.27 (0, 315.92), *p* = 0.93
High PA + Very High MD	4.44 (0.43, 47.55), *p* = 0.19	1.01 × 10^9^ (5.21 × 10^−104^, -), *p* = 1	0.01 (4.65 × 10^−7^, 14.76), *p* = 0.44
Very high PA + Low MD	5.63 (1, 45.85), *p* = 0.064	1.95 × 10^8^ (6.35 × 10^−100^, -), *p* = 1	0.23 (0, 70.91), *p* = 0.63
Very high PA + Medium MD	1.36 (0.18, 12.04), *p* = 0.76	4.29 × 10^7^ (4.13 × 10^−108^, -), *p* = 1	0.006 (8.43 × 10^−6^, 0.42), *p* = 0.048
Very high PA + High MD	1.38 (0.05, 17.65), *p* = 0.81	3.06 × 10^8^ (1.34 × 10^−97^, -), *p* = 1	1.69 × 10^−9^ (-, 1.06 × 10^174^), *p* = 1
Very high PA + Very high MD	2.94 × 10^−7^ (2.69 × 10^−302^, 5.69 × 10^24^), *p* = 0.99	0.3 (2.58 × 10^−72^, 3.64 × 10^70^), *p* = 1	2.95 × 10^−10^ (-, 3.65 × 10^212^), *p* = 1

Stratified multivariable logistic regression models, adjusted for maternal age, pre-pregnancy BMI (continuous), sedentary hours per day and frequency of post-lunch naps, smoking status, mode of conception, thyroid disorder, total energy intake. GDM: gestational diabetes mellitus; PA: physical activity; BMI: body mass index.

## Data Availability

Data are unavailable due to privacy restrictions concerning patient confidentiality.
